# The five-link theory for improving the integrated and balanced development of emergency medical care in urban and rural areas

**DOI:** 10.7189/jogh.15.03023

**Published:** 2025-07-01

**Authors:** Weifeng Shen

**Affiliations:** Department of Emergency Medicine, the Second Affiliated Hospital, Zhejiang University School of Medicine, Hangzhou, China

## Abstract

Over the past decades, considerable advancements have been made in China's emergency medical service system (EMSS). The disparity in EMSS development between urban and rural areas in China continues to be a significant public health concern. It also represents a pressing challenge on a global scale. Implementing strategies to narrow the urban-rural gap in emergency care and achieve balanced EMSS development holds critical significance. Therefore, this viewpoint presents the conceptual framework of the five-link theory and the five-link emergency care chain. The five-link emergency care chain constitutes the ground-breaking conceptual framework systematically addressing persistent challenges in achieving urban-rural health care integration and equitable advancement of emergency medical services. This framework constitutes a comprehensive EMSS, encompassing five core operational components: village (community) primary emergency care points, township emergency care units, prehospital emergency care, in-hospital emergency care, and critical care. It is engineered to optimise structural coherence, response efficacy, and service equity within EMSS. With the advancement of the five-link emergency care chain model implementation, its theoretical framework continues to evolve. This integrated theory-practice paradigm offers Chinese insights and experience in advancing global emergency medicine modernisation. Establishing comprehensive theoretical frameworks guiding emergency care service optimisation at township and village levels is imperative to facilitate the advancement of rural EMSS and mitigate urban-rural disparities in emergency medical services capacity. The five-link theory, grounded in five-link emergency care chain practices, offers a valuable framework for advancing urban-rural coordination of emergency care and ensuring equitable distribution of emergency response resources.

China's emergency medical service system (EMSS) has demonstrated substantial progress in recent decades, marked by rural-urban disparities in infrastructure development and emergency response capacities [1]. Rural EMSS is now a systemic vulnerability within the national emergency care framework. The significant gap in emergency medical services between rural and urban areas in China is mainly reflected in several aspects, which are structural inequities in EMSS resource allocation, policy and fiscal prioritisation gaps, workforce deficits and skill disparities, emergency response time disparity, unequal access to training and digital divide in emergency medical services [2]. The imbalance in EMSS development between urban and rural areas in China remains a prominent public health issue [3,4]. It is also a pressing global health challenge [5–10]. How can the urban-rural gap in emergency care be narrowed and balanced EMSS development achieved? The strategic enhancement of rural emergency medical infrastructure and capabilities, concurrently advancing equitable service delivery, bridging geographical disparities, constitutes a fundamental imperative for EMSS development. The global health community focusses on rural EMSS advancement via integrated strategies: optimised governance frameworks, infrastructure upgrading, specialised equipment distribution, health workforce capacity strengthening, and mobile medical unit deployment [11–14]. China's rural health care system faces structural challenges, characterised by extensive rural regions and a vast rural population coexisting with marked disparities in medical resource allocation [15,16], and structural inequalities persist in rural emergency care accessibility. China is entering a critical phase of systemic health care modernisation. Aligned with its national context, China strategically executes health policies focussed on sinking high-quality medical resources to grassroots health care tiers and promoting urban-rural and interregional equity in health care development [17].

The five-link emergency care chain constitutes the ground-breaking conceptual framework systematically addressing persistent challenges in achieving urban-rural health care integration and equitable advancement of emergency medical services. This framework constitutes a comprehensive EMSS, encompassing five core operational components: village (community) primary emergency care points, township emergency care units, prehospital emergency care, in-hospital emergency care, and critical care. It is engineered to optimise structural coherence, response efficacy, and service equity within EMSS. The five-link theory, grounded in the five-link emergency care chain practices, offers a valuable framework for advancing urban-rural coordination of emergency care and ensuring equitable distribution of emergency response resources. This viewpoint presents the conceptual framework of the five-link theory and the five-link emergency care chain.

## Origin of the five-link theory of emergency care

### Theoretical foundation

The current EMSS in China is generally constructed and operated based on the three-link theory, which has been proposed over two decades [18]. The three-link theory refers to three links of the basic framework of the EMSS, namely prehospital emergency care, in-hospital emergency care, and critical care [19]. These three links are interconnected as a whole body, constructing a continuum of care for critically ill emergency patients. This theory has contributed significantly to developing system frameworks, innovating models, enhancing capabilities, and optimising response times within urban EMSS in China, while remaining instrumental in advancing these critical domains. However, rural EMSS in China face system fragmentation in care coordination, urban-rural resource allocation gaps, and acute shortages in geographically isolated areas. As can be seen, the three-link theory does not have clear guiding objectives for constructing emergency medical services at the township and village levels. It lacks clarity in promoting the development goals of rural emergency care services and the concept of urban-rural integration development, and demonstrates limitations in addressing urban-rural emergency medical services disparities.

### The five-link theory

To facilitate the advancement of rural EMSS and mitigate urban-rural disparities in emergency medical services capacity, it is imperative to establish comprehensive theoretical frameworks guiding emergency care service optimisation at township and village levels. Building upon the foundational principles of the existing three-link theory, this paper demonstrates the five-link theory framework for emergency care combined with the practical experience of previous pilot projects and theoretical reasoning. The five-link theory guides the integration and development of the two grassroots links of township and village emergency care with the original three links of emergency care to form an integrated urban-rural EMSS. This theory will provide a theoretical foundation for facilitating the efficient allocation of high-quality emergency medical resources from urban to rural areas, establishing grid-based infrastructure for rural EMSS, while enhancing the coordinated response between prehospital and in-hospital emergency care and promoting operational integration within a hierarchical urban-rural emergency care framework, thereby elevating the overall service quality of regional emergency medical provision.

### The five-link emergency care chain

**Figure 1:**
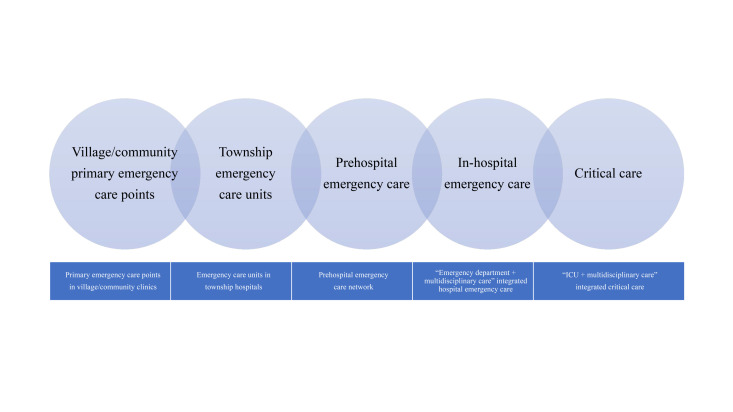
The five-link emergency care chain of village/community primary emergency care points, township emergency care units, prehospital emergency care, in-hospital emergency care, and critical care.

The five-link emergency care chain operationalises the five-link theory through a three-tiered expansion – prehospital, in-hospital, and intensive care – systematically extending to township and village (community) tiers, connecting township emergency care units and village (community) primary emergency care points, as shown in **Figure 1**. This unified emergency care framework establishes an urban-rural service integration ensuring equitable rural coverage, characterised by five system-driven attributes: operational timeliness, care integrity, service continuum, resource equilibrium, and universal accessibility. Initiated in a county of eastern China as a pilot, this project implements a tiered EMSS across the county, township, and village administrative tiers. Empirical evidence confirms the structural feasibility of county-level five-link emergency care chain development, with health system outcomes demonstrating progressive efficacy. Currently, the application of the five-link theory to practice remains in its early stages. Initial applications of the five-link theory and the five-link emergency care chain demonstrate enhanced EMSS distribution across urban and rural areas, improved operational efficiency, and strengthened emergency care competencies among health care professionals. Gradually, the five-link emergency care chain has been adopted in counties across a growing number of provinces in China, with its service coverage expanding to an increasing population.

### The basic components and functions of the five-link emergency care chain

The village (community) primary emergency care points constitute the primary tier in the five-link emergency care chain, functionally extending county-level hospital emergency departments and township emergency care units to village settings through integrated linkages with township emergency care units and prehospital emergency care networks. A village (community) primary emergency care point is designed to provide immediate response, rapid identification, initial disposal, and standardised patient transfer, enhancing the accessibility and efficiency of emergency medical services in rural communities. Typically located within village (community) clinics, these community-based emergency points enable trained medical personnel to deliver critical first-aid interventions to residents during medical emergencies. Equipped with mobile dispatch systems (smartphone apps), these health care responders can rapidly reach emergency scenes upon receiving real-time alerts from emergency response centres and residents. Crucially, village primary emergency care personnel serve as vital frontline responders during the critical prehospital gap, delivering time-sensitive emergency interventions for patients experiencing acute life-threatening conditions before ambulance arrival in remote regions. Moreover, village primary emergency care points function as rural training centres for community members and lay responders, empowering local volunteers to administer critical cardiac emergency interventions during the pre-ambulance phase of out-of-hospital cardiac arrests [20–22].

The township emergency care units serve as the secondary link of the five-link emergency care chain, extending services from county-level hospital emergency departments to townships. As a coordinating hub, it systematically organises emergency care delivery across county-township-village administrative divisions through standardised operational protocols. The township emergency care unit, typically in township hospitals, integrates multidisciplinary emergency specialities. Key components include trauma management, chest pain intervention, stroke care, critical obstetrics and neonatal emergency services, and dedicated resuscitation/shock treatment facilities. Its subspecialty configuration features scalable functionality, enabling modular expansion according to evolving clinical needs and regional health care priorities. The township emergency care unit operates via a framework covering early identification, initial stabilisation, and protocol-driven transfer of critical cases, adhering to standardised emergency protocols and speciality care guidelines. It is recognised that establishing and sustaining township emergency care units necessitate addressing systemic challenges. Practically, implementation should follow phased development: first deploying time-critical priority specialities (*e.g.* chest pain, stroke, and trauma units), then expanding other subspecialties as clinically warranted. Establishing township emergency care units and rural emergency care points requires phased system integration with tiered capacity-building frameworks. This involves strategic resource allocation across rural health ecosystems to strengthen frontline acute care capabilities while improving infrastructure readiness in primary care settings through prioritised expansion of emergency medical services. This strategic framework guides the phased development of specialised emergency care units in township hospitals, integrating rural health system enhancements to strengthen acute care delivery in primary settings. Effective implementation requires multifaceted support, including sustained funding and prioritised investment in workforce development through continuous education and training, which are critical drivers for expanding rural emergency care capacity [23]. China's national policy prioritises enhancing grassroots health care capabilities to optimise primary health care services [24]. The ongoing rural health care modernisation drive strategically allocates resources to bridge the urban-rural gap in medical service quality.

Prehospital emergency care constitutes the pivotal linkage in emergency care systems, connecting village-level emergency care points, township-level emergency care units, and county-level hospital emergency departments into a coordinated care network. Emergency medical services should implement a balanced regional development framework, systematically establishing county-wide prehospital networks through coordinated county-level emergency centres and full township-level station coverage [25]. Furthermore, township-level ambulance deployment requires intensification while maintaining urban-rural resource equity to reduce emergency medical services response times and shorten transfer duration.

In-hospital emergency care serves as a critical nexus bridging prehospital emergency care, in-hospital specialities, and intensive care treatment, while functioning as a pivotal hub for extending emergency medical resources to the township emergency care units and the village (community) primary emergency care points. Building upon a seamless transition between prehospital and in-hospital emergency care, an integrated ‘emergency medicine + multidisciplinary collaborative care’ model is established to deliver efficient, protocol-driven emergency diagnosis and treatment. Optimising the prehospital time interval and ensuring timely delivery of definitive care for critically ill patients are critical to improving survival outcomes in time-sensitive emergencies. Hospital emergency departments systematically enhance efficiency and comprehensive care delivery through infrastructure enhancement, spatial optimisation, process streamlining, and specialised team development. This strengthens county-level hospital emergency departments as pivotal clinical hubs within tiered emergency care systems.

Critical care operates as a key link in the continuum of care for critically ill patients in the five-link emergency care chain. Implementing integrated emergency-critical-operative care systems should be accelerated, while strengthening multidisciplinary care coordination across the critical care continuum [26]. Critical care outreach and borderless care models drive system-level advancement in emergency-critical care through enhanced cross-sector coordination [27]. According to the treatment needs of critically ill patients, it is highlighted that critical care technologies need to be gradually advanced to upstream tiers of the five-link emergency care chain, particularly during prehospital and initial phases. To enhance the capacity and resilience of the five-link emergency care chain, it is recommended to explore the strategic deployment of mobile intensive care units in prehospital settings and adaptable intensive care unit surge capacity within hospitals.

## Operation and development of the five-link emergency care chain

### Synergy and integrated operations of the five-link emergency care chain

China's county-level health care systems implement a medical consortium model integrating county hospitals, township hospitals, and village clinics under unified governance. This tiered framework prioritises centralised management, resource sharing, and digital system integration. County-level five-link emergency care chain pilots establish interoperable information networks through a triad of digital solutions: medical priority dispatch system, emergency response apps, and prehospital-hospital care coordination systems. The local health office manages and coordinates the logistics plans and the operations of the five-link emergency care chain. The local emergency medical quality control centre oversees the quality and safety across the emergency care chain. At the county level, the efficiency of the five-link emergency care chain relies on standardised frameworks that integrate prehospital-hospital care coordination and establish three-tier county-township-village response networks.

### Challenges and solutions of the integration of digital emergency care in rural areas

Regional pilot programs have implemented a county-wide integrated digital platform enabling coordinated prehospital-hospital emergency care. This interoperable health information exchange system facilitates key data sharing between urban and rural health care facilities. The prehospital emergency centre activated ambulance deployment and mobilised tiered responders via intelligent dispatch platforms upon emergency verification. This real-time coordination engaged township emergency care units, village emergency care points, and certified social first-aid volunteers to deliver rapid on-site care before ambulance arrival. The strategic implementation of fifth-generation wireless networks and next-generation digital infrastructure across China has created an intelligent connectivity framework for grassroots-level high-speed data dissemination [28]. In rural China, propelled by national strategic initiatives like rural revitalisation [29,30], the government-supported medical education program for rural-oriented talent development has steadily expanded. This has driven a marked increase in young health care professionals, while the digital literacy gap among medical personnel is being notably addressed through growing technological adoption across the health care ecosystem.

### Challenges and solutions for scaling up emergency care in resource-constrained rural areas

Enhancing emergency medical services in rural settings necessitates prioritising resource allocation as a critical determinant. Establishing the five-link emergency care chain requires strategically redistributing high-quality emergency care infrastructure to rural and community-based facilities, fostering equitable inter-regional distribution of emergency medical resources and effectively mitigating urban-rural disparities in acute care accessibility. In China, universal institutional coverage of health care infrastructure has been established across all counties, townships, and villages under the Healthy China 2030 initiative, forming a comprehensive service network that aligns with the three-tier administrative hierarchy of rural governance systems [31]. Strategically deployed mobile health units enhance resource efficiency in remote, resource-limited areas while establishing critical emergency care networks for underserved communities. Advancing the five-link emergency care chain requires strategically integrating telemedicine, mobile health units, and partnerships with urban hospitals within health policy frameworks. This integrated approach enhances rural emergency medical service efficiency while strengthening urban-rural emergency care coordination in resource-limited settings [32–35].

### Critical factors influencing the five-link emergency care model implementation in rural settings

Social and cultural factors significantly influence the evolution and functioning of the five-link emergency care chain. Cultural compatibility is a key determinant in contextualising emergency protocols, with more substantial alignment to local contexts correlating directly with implementation efficacy. The effective deployment of the five-link emergency care chain requires strategic planning and targeted promotion, particularly when addressing social and cultural influences within communities. The prioritisation of rural emergency response infrastructure development and the mitigation of the urban-rural gap in acute care accessibility constitute a critical determinant in the operationalisation and systematic scaling-up of the five-link emergency care chain under the health policy framework. Government-led initiatives strengthening primary care infrastructure have bolstered public confidence in health care systems [36], with patients increasingly seeking treatment at community-level facilities [37]. Increasing trust in the health care system improves the utilisation rate of grassroots emergency networks and brings more benefits to developing grassroots emergency care. Similarly, the effectiveness of grassroots emergency care is conducive to building trust in the health care system. Traditional Chinese medicine holds a broad foundation with enduring cultural affinity in China's rural regions, positioning it as a demand-responsive mechanism to strengthen health system resilience. This endogenous health care asset offers strategic complementarity to medical emergency response frameworks, particularly where conventional medical infrastructure remains limited.

### Sustainable financing mechanisms for emergency care scale-up in resource-constrained rural health care systems

The operationalisation and systematic expansion of the five-link emergency care chain necessitate robust financial undergirding. Persistent systemic capacity deficits in rural acute care delivery systems, coupled with pronounced socio-spatial disparities in emergency medical service accessibility, mandate the development of evidence-based intervention strategies through multi-stakeholder collaboration. Under circumstances characterised by constrained emergency medical resource availability, the five-link emergency care chain emerges as a viable operational strategy that facilitates the systematic extension of urban EMSS to township and village-level health care settings. There is a compelling need to institute policy frameworks that enable the strategic allocation and vertical integration of high-calibre emergency care resources from urban centres to rural communities, thereby addressing the existing disparity in resource distribution patterns. The five-link emergency care chain development should be strengthened to optimise the allocation of high-quality emergency medical resources from urban to rural areas through urban-rural collaboration mechanisms. The five-link emergency care chain model demonstrates superior cost-effectiveness compared to exclusive dependence on governmental funding for rural emergency care systems, particularly in operational efficiency and resource utilisation outcomes, while maintaining the necessity for fiscal allocations to underserved rural regions. In China, specifically, support mainly comes from three aspects: direct fiscal allocations by governments at all levels, county-level medical consortia implement structured resource integration mechanisms, concurrently advancing health system consolidation and clinical workforce development, and provincial/municipal tertiary hospitals deliver institutionalised counterpart assistance through technical guidance and competency-aligned training systems. The EMSS in China operates under a public welfare paradigm, predominantly funded through government-led infrastructure investments, while strategically incorporating public-private partnerships for system development. Regions demonstrating robust governmental fiscal commitments exhibit enhanced developmental trajectories in rural EMSS, particularly in mitigating urban-rural disparities. Strategic advancement of the five-link emergency care chain necessitates establishing multidimensional financing architectures that synergise public-sector allocations, cross-sector partnership models, and innovative financing mechanisms. Resource-scarce rural regions should prioritise mobilising targeted government grants and anti-poverty social welfare allocations through structured fiscal coordination mechanisms.

## Prospects for the development of the five-link emergency care chain

### Policy support and direction of health care reform

China's health equity agenda prioritises rural populations through enhanced primary care systems, as mandated by the Outline of the Healthy China 2030 Plan [38]. Urban-rural disparities in health care access and health outcomes have achieved measurable reductions via equity-driven resource redistribution to health-disadvantaged areas. In recent years, China's national health policy framework has prioritised strategic initiatives encompassing the equitable distribution of high-quality medical resources, optimisation of spatial health care allocation, implementing a hierarchical medical system, and capacity-building for primary care institutions. Concurrently, China's national strategic initiatives, exemplified by rural revitalisation, have established an enabling policy infrastructure for operationalising the five-link emergency care chain. The policy directives promulgated by China's National Health Commission emphasise the imperative to enhance infrastructure and service delivery mechanisms at township hospitals and village clinics, while facilitating systemic integration across urban-rural dimensions to optimise service delivery capabilities at grassroots health care facilities [39]. This policy framework provides substantive institutional support for implementing and scaling of the five-link theory model. The practice of the five-link emergency care chain model can be carried out in stages. The county-level empirical implementation provides a foundation for expanding the scope of multitiered EMSS across provincial-municipal-county-township-village hierarchies, with concurrent advancement toward establishing a stratified, functionally differentiated, and systemically resilient five-link emergency care chain through comprehensive institutional optimisation.

### The significance of advancing paradigm innovation in global EMSS

In countries with large rural populations and vast territories, implementing EMSS in non-urban areas faces systemic challenges. These include inefficient time-space coordination during emergency responses, severe shortages of specialised emergency medical staff, and inadequate critical life-support facilities [40,41]. The five-link theory framework provides philosophical insights and practical solutions for addressing global health issues. While remote and mobile health care are supplementary approaches to enhance EMSS in rural and geographically remote areas [42–44], especially under resource-scarce conditions, they cannot replace permanent infrastructure or systemic solutions. The five-link emergency care chain could serve as a sustainable strategy to systematically extend urban-grade EMSS to rural and remote areas through five key measures: strategic emergency resource decentralisation, coordinated urban-rural development, deployment of qualified emergency personnel, essential medical infrastructure establishment, and progressive enhancement of rural emergency response capacities. This approach aims to reduce urban-rural EMSS disparities through phased implementation. The process requires integrating permanent frameworks with interim measures through structured processes, allowing for mutual reinforcement and continuous improvement.

### Applicability and replication of innovative models

The five-link emergency care chain enhances system integration between urban and rural areas through coordinated resource distribution and standardised protocols, to address emergency response capability and efficiency disparities. This model is particularly relevant for regions seeking to bridge the urban-rural health care gap and achieve balanced EMSS development. However, implementation requires careful consideration of regional variations in health care needs, infrastructure readiness, and governance structures. Regional health priorities and health care system readiness provide practical foundations for implementing the five-link emergency care chain model. Applying this framework requires evidence-based adaptations that systematically integrate socio-ecological determinants and multi-stakeholder governance, ensuring compatibility with local health care ecosystems. For practical execution, regions should formulate phased implementation strategies that align resource allocation with defined objectives and regional capacity assessments.

## CONCLUSIONS

The five-link theory and the five-link emergency care chain represent groundbreaking innovations in advancing health care equity and emergency care accessibility across China's urban-rural continuum. While this initiative remains promising, it requires further practical validation and refinement. These systemic improvements will drive structural integration of EMSS, systematically bridging urban-rural disparities in emergency response capabilities while ensuring equitable access to high-quality emergency care for all populations. With the advancement of the five-link emergency care chain model implementation, its theoretical framework continues to evolve. This integrated theory-practice paradigm offers Chinese insights and experience in advancing global emergency medicine modernisation through context-specific adaptations in health governance.
